# Novel *GLI3* variant causing overlapped Greig cephalopolysyndactyly syndrome (GCPS) and Pallister-Hall syndrome (PHS) phenotype with agenesis of gallbladder and pancreas

**DOI:** 10.1186/s13000-017-0682-8

**Published:** 2018-01-03

**Authors:** Saki Ito, Riko Kitazawa, Ryuma Haraguchi, Takeshi Kondo, Ayaka Ouchi, Yasuo Ueda, Sohei Kitazawa

**Affiliations:** 1 Department of Molecular Pathology, Ehime University Graduate School of Medicine, Shitsukawa, Toon City, Ehime 791-0295 Japan; 2 Division of Diagnostic Pathology, Ehime University Hospital, Toon City, Ehime 791-0295 Japan; 3 Division of Legal Medicine, Kobe University Graduate School of Medicine, Kobe City, Hyogo 650-0017 Japan

**Keywords:** GLI3, Hedgehog, Greig cephalopolysyndactyly syndrome, Pallister-hall syndrome

## Abstract

**Background:**

A proper balance between the activator and the repressor form of GLI3, a zinc-finger transcription factor downstream of hedgehog signaling, is essential for proper development of various organs during development. Mutations in different domains of the GLI3 gene underlie several congenital diseases including Greig cephalopolysyndactyly syndrome (GCPS) and Pallister-Hall syndrome (PHS).

**Case presentation:**

Here, we describe the case of an overlapped phenotype of these syndromes with agenesis of the gallbladder and the pancreas, bearing a c.2155 C > T novel likely pathogenic variant of GLI3 gene by missense point mutation causing p.P719S at the proteolytic cleavage site.

**Conclusions:**

Although agenesis of the gallbladder and the pancreas is uncommon in GLI3 morphopathy, a slight difference in the gradient or the balance between activator and repressor in this case may hinder sophisticated spatial and sequential hedgehog signaling that is essential for proper development of gallbladder and pancreas from endodermal buds.

## Background

GLI3 is a zinc-finger transcription factor that mediate the sonic hedgehog (SHH) signaling acting as both a transcriptional activator and repressor during development [1, 2]. When the SHH is present, full-length GLI3 up-regulates its target genes, whereas when the SHH is absent, GLI3 is cleaved to yield a repressor that down-regulates those genes [3]. The pathogenic variants of GLI3 gene cause various malformations including Greig cephalopolysyndactyly (GCPS) syndrome [2] and Pallister-Hall syndrome (PHS) [4] in an autosomal dominant pattern. Although GCPS and PHS have some distinct clinical presentations (GCPS is characterized by polysyndactyly, macrocephaly, hypertelorism, and PHS is characterized by hypothalamic hamartoma, bifid epiglottis, and insertional polydactyly), the apparent lack of GLI3 genotype-phenotype correlation occasionally precludes clear phenotypic classification [5]. Described here is a case of an overlapped GCPS and PHS phenotype with agenesis of the gallbladder and the pancreas, bearing a novel likely pathogenic GLI3 variant by point mutation.

## Case presentation

The 31-year-old woman, gravida-1 para-1, with no apparent risk factors for congenital anomaly, achieved natural pregnancy. Oligohydramnios and intrauterine growth restriction was, however, noted at 14 weeks of gestation. Amniocentesis, carried out at 16 weeks of gestation for chromosomal analyzes, revealed a normal 46, XY male karyotype pattern. At 30 weeks of gestation, echographic examination revealed loss of fetal movement; intrauterine fetal death was confirmed 3 days thereafter, and the fetus was delivered by artificial abortion.

### Autopsy findings

**Fig. 1:**
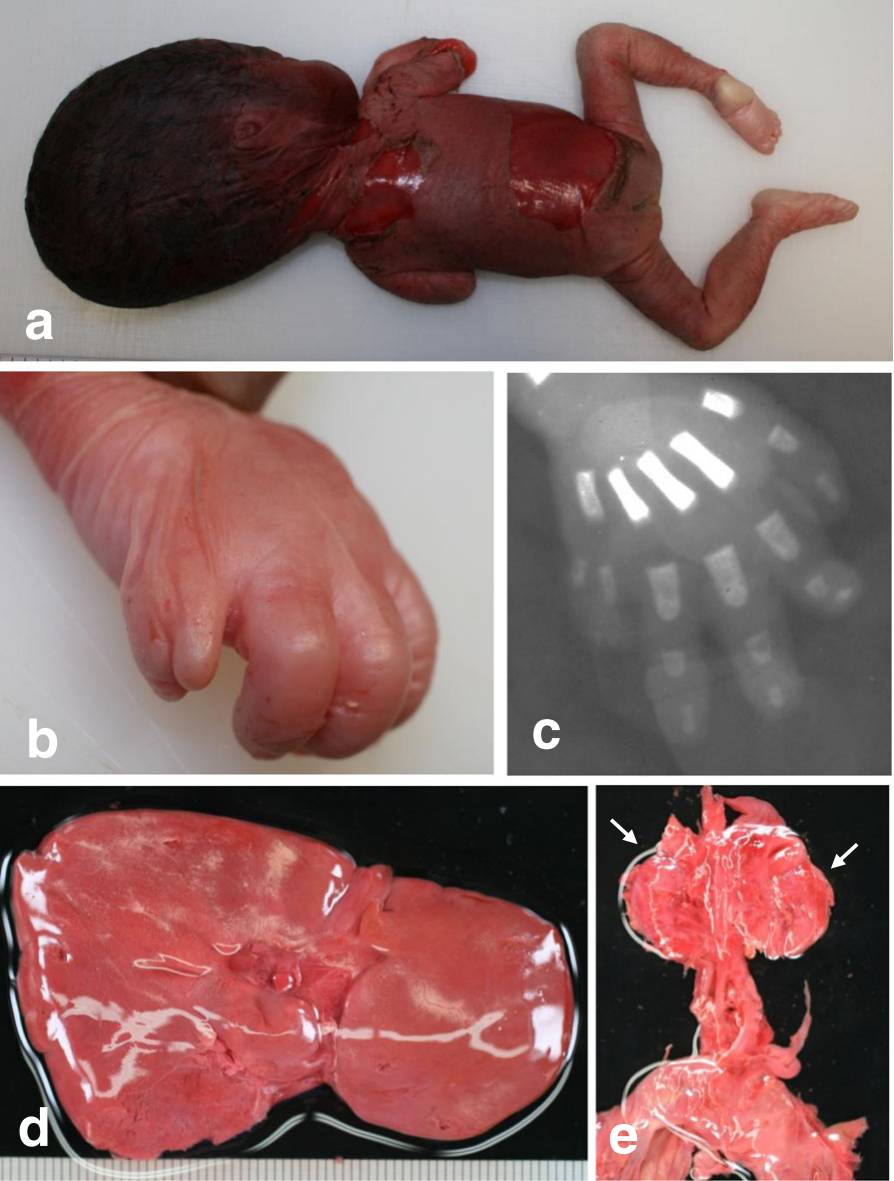
Macroscopic findings at autopsy. The stillborn had a proportionally unbalanced, large head with acrocephaly (**a**). Postaxial polysyndactyly of the right hand was noted (**b**) and (**c**, photography by soft X-ray). While the shape and size of the liver were normal, the gallbladder was nonexistent at the hepatic portal section (**d**, hatched area). Hypoplasia of both kidneys (**e**) was noted (arrows)

**Fig. 2:**
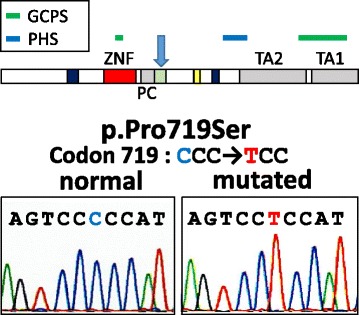
PCR was conducted with the use of agarose beads from paraffin sections as templates. Each PCR product was cloned into TA-vector, and amplified plasmids were analyzed for the DNA sequence. A missense mutation (c.2155 C > T) leading to p.P719S was detected in 6 of 12 independent clones from PCR targeting exon 6. By this missense mutation, an amino acid property is changed from hydrophobic (Proline, P) to hydrophilic (Serine, S) within the proteolytic cleavage (PC) site of GLI3 (blue arrow in upper panel). Typical location of pathogenic variants of GLI3 gene for GCPS (green bar) and PHS (red bar) are illustrated according to the report by Demurger F et al. [19]

Autopsy carried out 2 h after delivery of the 600 g stillborn revealed multiple systemic malformations. Macroscopic autopsy findings are summarized in Table 1. The stillborn demonstrated a proportionally unbalanced, large head with acrocephaly (Fig. 1a), postaxial polysyndactyly (Fig. 1b and c), gastrointestinal malformations including malrotation and atresia of the anus, agenesis of the gallbladder (Fig. 1d) and the pancreas, hypoplasia of both kidneys (Fig. 1e), and the endocrine organs. Except these malformations, histopathological alteration of other major organs such as heart, liver, and bone was not noted. In view of these macroscopic features suggestive of GCPS or PHS, mutation of the GLI3 gene was analyzed. Routinely formalin-fixed (10%) and paraffin-embedded archival samples of infant thymus tissue samples obtained from Kobe University Hospital (Kobe, Hyogo) deparaffinized with xylene, suspended in 5 μl of 1 × TE and then mixed with pre-warmed and liquefied low-melting agarose (3.2%) at 1:1. A total of 10 μl of agarose beads containing 1 × TE and tissue fragments was formed in 250 μl of pre-chilled mineral oil, and then incubated at 50 °C overnight in 1000 μl of 200 μg/ml proteinase K, 10 mM Tris-HCl (pH 8.0) and 25 mM ethylene diamine tetraacetic acid. Bead fragments were washed in 1 × TE, sliced into several pieces, and analyzed by PCR using sets of primers encompassing all known coding exons and exon–intron boundaries of the GLI3 gene. Primers were designed as described [6]. The PCR mixture contained Mighty AMP® DNA polymerase (Takara, Tokyo, Japan) and bead fragments in a final volume of 25 μl. The PCR products were electrophoresed in a 3% agarose gel and stained with ethidium bromide. Purified PCR products were cloned into TA-vector, and amplified plasmids were analyzed for the DNA sequence. Each PCR product was analyzed by sequencing, and the missense mutation (c.2155 C > T) leading to p.P719S was detected in 6 of 12 independent clones from PCR targeting exon 6, which altered the amino acid property from hydrophobic (Proline, P) to hydrophilic (Serine, S) within the proteolytic cleavage (PC) site of GLI3 (Fig. 2). Because this missense mutation was absent in both parents (data not shown), without family history of GCPS or PHS, and non-maternity factors like egg donation, surrogate motherhood, and errors in embryo transfer, the c.2155 C > T mutation probably occurred de novo in the fetus, albeit parental germline mosaicism cannot be ruled out.

**Table 1:** Macroscopic findings

Endocrine organs	Thyroidal atrophy, adrenal atrophy
Digestive organs	Malrotation of intestine, atresia of anus, agenesis of gallbladder, agenesis of pancreas
Urinary organs	Bilateral hypoplasia of kidney
Genitals	Bilateral cryptorchidism, external genitalia hypoplasia, and hypospadias
Others	Postaxial polysyndactyly, and polysplenia,

## Discussion

GLI3 comprises several functional domains: repressor domain (RD, aa106-aa263), zinc-finger DNA binding domain (ZNF, aa462-aa645), proteolytic cleavage site (PC, aa703-aa740), CREB binding protein domain (CBP, 827-1131), transactivation domain 2 (TA2, aa1044-aa1322) and transactivation domain 1 (TA1, aa1376- aa1580), [7, 8]. GLI3 has a dual function as a transcriptional activator and a repressor of the SHH pathway — the full-length GLI3 acts as an activator after phosphorylation and nuclear translocation, while its C-terminally truncated form by protease clearage acts as a repressor [3, 9, 10]. A proper balance between the GLI3 activator and the repressor, organized by hedgehog signaling, elicits proper evolvement of the various organs during development [11–13]. Pathogenic variants in different domains of the gene underlie several congenital diseases including GCPS (MIM ID#175700), PHS (MIM ID #146510), preaxial polydactyly type IV (MIM ID #174700), and postaxial polydactyly types A1 and B (MIM ID #174200), Acrocallosal Syndrome (MIM ID #200990), trigonocephaly with craniosynostosis and polydactyly, and some types of oral-facial-digital syndromes [14]. Typical GCPS involves polysyndactyly of hands and feet, and craniofacial abnormality. Different genetic alterations (from chromosomal deletion to a single nucleic acid mutation in the ZNF domain that reduces the functioning of both the activator and repressor forms of the GLI3 protein) cause GCPS [15]. PHS, on the other hand, shows a lethal condition involving hypothalamic dysfunction, skeletal limb defects, craniofacial and urogenital malformation attributed to pathogenic variants of GLI3 gene, typically within or around the PC domain of the GLI3 protein, resulting in a relative reduction in the functioning of the GLI3 repressor form [16]. Since a delicate balance between the functioning activator and repressor is essential for proper development [2, 17, 18], the simple location of the pathogenic variants in GLI3 site does not always clearly correlate with the clinical manifestations [19]. Also, some pathogenic variants cause overlapping phenotypes of GCPS and PHS [19]. Therefore, the concept of GLI3 morphopathies has been postulated to designate these mutually overlapped syndromes [5].

In the current study, we demonstrated a case of GLI3 morphopathy attributed to a novel likely pathogenic variant by missense mutation at the PC domain (p.P719S) of GLI3 (variant was judged as likely pathogenic based on the criteria by American College of Medical Genetics and Genomics and the Association for Molecular Pathology, ACMG standards and guidelines [20]). Indeed, in silico predictions of the newly found variant (p.P719S) of GLI3 gene via the predictive algorithms (PolyPhen-2, http://genetics.bwh.harvard.edu/pph2) shows that this mutation is predicted to be “PROBABLY DAMAGING” with a score of 0.963 (sensitivity: 0.78; specificity: 0.95).Interestingly, besides gastrointestinal malformations, agenesis of both the gallbladder and the pancreas was observed. During the early stages of organogenesis, the liver, the gallbladder and the biliary duct system arise as buds in the endodermal epithelium at the distal end of the foregut. A small caudal portion of the liver bud then expands to form the gallbladder [21]. The pancreas develops also from the endodermal epithelium of the duodenal diverticula, initially as two separate dorsal and ventral pancreatic buds, which then fuse when the duodenum rotates [22]. SHH signaling is expressed in the ventral foregut endoderm from which the liver derives, and its expression disappears at the onset of liver bud formation [21, 22]. Such sophisticated spatial and sequential SHH signaling is essential for proper organ development from these endodermal buds. Because we have not done comprehensive genomics study, possibility of coincidental additional pathogenic variants in Pdx1, Ptf1a, Gata4 and Gata6, known to be associated with agenesis of pancreas [23], cannot be excluded in this case. Although it remains unclear why agenesis of the gallbladder and the pancreas is uncommon in GLI3 morphopathy, a slight difference in the gradient or the balance between activator and repressor induced by a slight difference of protein tertiary structure may contribute to the apparent phenotypic difference in GLI morphopathy.

## Conclusion

In conclusion, this case is an extreme example in which slight difference of genetic alterations in the same gene may result in considerable morphological variations, when sophisticated spatial and sequential expression of the gene is essential and critical for proper development.

## Abbreviations

CBP: CREB binding protein domain
GCPS: Greig cephalopolysyndactyly syndrome
PC: Proteolytic cleavage site
PCR: Polymerase chain reaction
PHS: Pallister-Hall syndrome
SHH: Sonic hedgehog
TA: Transactivation domain
ZNF: Zinc-finger DNA binding domain
